# DMF Index among Amelogenesis Imperfecta Patients: Systematic Review of the Literature

**DOI:** 10.1155/2021/5577615

**Published:** 2021-08-17

**Authors:** Farah Kallel, Amel Labidi, Sana Bekri, Sinda Ammar, Sonia Ghoul, Lamia Mansour

**Affiliations:** ^1^Partial Removable Prosthetic Department, Faculty of Dental Medicine, University of Monastir, Avicenna Avenue, Monastir 5019, Tunisia; ^2^ABCDF Laboratory for Biological, Clinical and Dento-Facial Approach, University of Monastir, Avicenna Avenue, Monastir 5019, Tunisia; ^3^Laboratory of Histology and Embryology, Faculty of Dental Medicine, University of Monastir, Avicenna Avenue, Monastir 5019, Tunisia

## Abstract

**Objectives:**

The aim of this study was to explore the literature in order to assess systematically the association between amelogenesis imperfecta (AI) and caries development and to evaluate the DMF index among AI patients. *Basic Research Design*. PubMed was used to explore the database Medline. The key words used were “Amelogenesis Imperfecta” [Mesh], “Dental Caries” [Mesh], “Tooth Loss” [Mesh], “DMF Index” [Mesh], and “Dental Restoration, Permanent” [Mesh]. Moreover, an ad hoc search was performed in order to make the study as exhaustive as possible.

**Results:**

Fifty-five articles were retained. The total number of patients gathered was 499. A percentage of 68.8% of the articles dealt with cases with a relatively low dental caries process, 20.8% dealt with cases in which the dental caries process was relatively moderate, and 10.4% dealt with cases in which the dental caries process was severe. Teeth extraction due to dental caries was mentioned in 10 articles. Eleven articles, concerning 53 patients, mentioned dental fillings. Four patients did not have dental filling due to dental caries. DMF index was very low in 2 articles and low-to-high in 3 articles.

**Conclusion:**

Low dental caries susceptibility with AI patients was noticed in this study. A possible factor could be the lack of proximal contacts and elimination of fissures through enamel loss. The lack of dental caries susceptibility was also explained by the microbacterial specificity of hypoplastic AI patients. Moreover, it was also noted that the prevalence of dental caries among AI patients depends on sociodemographic change.

## 1. Introduction

Amelogenesis imperfecta (AI) is a disorder of tooth development. This pathology causes alteration of the enamel structure that leads to discolored, pitted, grooved, or small teeth. Moreover, it leads to rapid wear of dental tissues and breakage. AI affects both primary and permanent teeth. In addition to that, it can occur either alone with no other signs or symptoms or as part of a syndrome that affects multiple parts of the body [1].

The decayed, missing, filled (DMF) index is established as the key measure of caries experience in dental epidemiology. It is called DMFT, when applied to tooth, and DMFS when applied to tooth surfaces. When written in lowercase letters, the dmf index is a variation that is applied to the primary dentition [2, 3].

Despite the structural abnormalities of the enamel that leads to the logical thinking that these teeth are more likely to develop dental caries, clinical observation reported in a recent article a low susceptibility of dental caries among AI patients [4].

The aim of this study was to assess the association between AI and caries development evaluating systematically the decayed, missing, filled (DMF) index in the literature among AI patients and to compare it to patients without AI.

## 2. Methods

### 2.1. Information Search and Sources

A systematic review of the literature exploring the Medline database was performed using PubMed. The search strategy was developed using the Mesh words “Amelogenesis Imperfecta” [Mesh]; “Dental caries” [Mesh]; “Dental Restoration Permanent” [Mesh]; “Dental loss” [Mesh]; “Dental caries” [Mesh], and “DMF index” [Mesh]. These terms were associated into different Boolean formulas (Table 1). An ad hoc search on Google Scholar and a manual research were also performed.

### 2.2. Study Eligibility Criteria

The literature research was started on November 2019, and the final update of the search was checked on February 2020. Criteria for articles' selection were defined before beginning the article screening. There was no restriction on the articles' type of study. However, articles which fall in any of the below categories were excluded.

- The article was not published in French or English
- The article does not treat the subject of AI
- The article does not mention dental caries, dental loss, or dental filling caused by caries

### 2.3. Data Collection

Data were collected by three independent reviewers using a preestablished checklist for data collection. In case of disagreements, consensus was achieved by discussion among the reviewers.

## 3. Results

### 3.1. Articles' Selection

**Figure 1:**
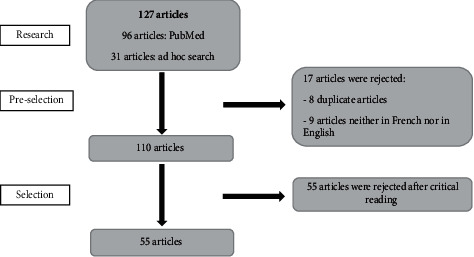
Flow diagram showing the search strategy.

The bibliographic search on MEDLINE, using keywords mentioned above without activating any selection filters, allowed to identify 96 articles (Table 1). Among the found studies, 72 were excluded as follows: 9 of these were excluded due to the fact that they were not published in French or English, 8 articles were identified as duplicates, and 55 articles did not explore the DMF index within AI patients. Moreover, based on an ad hoc search, 31 articles related to this study were found and hence retained. Thus, 55 articles in total were used in this study (Figure 1). Concerning the study design of the studies, 39 articles were case reports, 8 were cross-sectional studies, 6 were case series, 1 was a clinical trial, and 1 was a systematic review of the literature.

### 3.2. Patients' Characteristics

In the 55 selected articles, a total of 499 patients were described. The age of 5 patients was not specified in the articles, 492 patients had an age less than or equal to 28, and 2 patients had an age above 28 years. Among the studied population, 144 were female and 128 were male. The gender of 227 patients was not defined. AI types of the 499 patients studied were collected and are included.

- 153 patients with hypoplastic AI
- 100 patients with hypomatured AI
- 30 patients with hypocalcified AI
- 60 with hypoplastic/hypomatured AI associated with taurodontism
- 3 patients with hypoplastic/hypocalcified AI
- 9 patients were classified into a group of hypomineralized AI, which includes hypomatured and hypocalcified types
- 144 patients in 9 articles did not give details about AI type

One hundred and thirty-nine (139) patients had permanent dentition, 5 had temporary dentition, and 22 had mixed dentition. Dentition type was not specified for 333 patients.

### 3.3. Dental Caries

Seven articles among the 55 studied articles did not mention dental caries. The remaining 48 articles were divided into 3 groups (group A, group B, and group C).

Group A included 33 articles representing 68.8% of the retained articles. It dealt with cases with a relatively low dental caries process. In this group, the majority of the patients mentioned in the case report and case series were carious free or had a maximum of 2 dental decays. One patient, who was carious free, had seen the occurrence of small interdental caries after performing composite restorations for orthodontic treatment.

In 3 cross-sectional studies and a case series treating 183 patients, a low susceptibility and a low progression of dental caries was found.

The prevalence of caries in the follow-up period after prosthodontic treatment was found relatively low in 10 articles that included 157 patients. The rate of failure due to secondary caries was lower for patients affected by AI than for not affected patients.

Group B included 10 articles representing 20.8% of the retained articles. It dealt with cases in which the dental caries process was relatively moderate. This group included patients who had dental caries between 3 and 5. In the cross-sectional study by Adorno et al. [5] performed in Chile, AI patients had a similar number of tooth decay compared to healthy Chilean children. This particular study has shown that patients having hypoplastic AI had the fewest rates of dental caries.

Group C included 5 articles representing 10.4% of the retained articles. It dealt with cases in which the dental caries process was severe. Severe dental caries, greater than or equal to 6, and secondary caries were noted in 5 case reports. Two patients in this group suffered from AI associated to syndromes which are epidermolysis bullosa and bilateral nephrocalcinosis.

### 3.4. Tooth Loss

Teeth extraction due to dental caries was mentioned in 10 articles including 13 patients.

For 6 patients, a maximum of 2 permanent teeth were extracted. For 2 patients, 3 permanent teeth or more were extracted. For 3 patients, only primary teeth were extracted. Finally, for 2 patients, 2 permanent molars and lacteal teeth were extracted.

### 3.5. Dental Filling

Eleven articles, concerning 53 patients, mentioned dental fillings. Three patients did not have dental filling due to dental caries. The other patients had different types of fillings. They were mostly amalgam, glass ionomer cement, and composite resin.

A patient described in Light and colleague's case series had amalgam in 4 molars. Previous orthodontic banding had initiated the caries on those teeth [6].

Restorations caused for reasons other than dental caries were not recorded.

### 3.6. DMF Index

DMF index was mentioned in 6 articles and ranged from very low to high.

In the following 2 articles, the DMF index was evaluated to very low.

- A case series from Light et al. that dealt with 2 American patients for which the DMF index was noticed as “extremely low” [6]
- A cross-sectional study established by Kammoun et al. in Tunisia that dealt with 14 patients with AI. In this group, the average DMFT scored a value of 0.8 as opposed to 2.9 for a control group of the same number of patients [4].

In a Swedish cross-sectional study by Pousette et al., the DMF index was evaluated to low. The average DMFS value was found to be 2.5 +/− 4.1 for 82 Swedish patients with AI compared to a DMFS of 0.8 +/− 1.8 for a control group [7].

In a Turkish cross-sectional study by Koruyucu et al., which dealt with 31 patients, the DMFT average was found to be 2.74 +/− 1.71 and was considered moderate [8].

Markovic et al. conducted a cross-sectional study in Serbia on 12 patients. The DMF was evaluated as high [9].

Moreover, Sundell et al. performed a cross-sectional study that dealt with 105 patients in Switzerland. In this study, the number of decayed and filled proximal surfaces was found to be 3.7 in the hypoplastic type and 17.1 in the hypomineralized type of hereditary AI [10].

## 4. Discussion

Case reports and case series articles represented 81.5% of the total number of articles used in this study. This high number of case reports and case series could be attributed to the fact that AI is a rare disease and therefore cumulating a big sample of patients for clinical studies is difficult. In fact, Crawford et al. estimated the prevalence of AI patients to vary between 1/700 and 1/14000 [11].

In this study, the majority of the considered patients were relatively young, with an age range under 29. As stated in the literature, patients with AI tend to consult their dentist precociously. Treatment of this kind of disease is important especially for esthetic reasons and functional rehabilitation [9, 12–14]. Moreover, this treatment is considered mandatory to prevent further damage due to occlusal wear and disturbance of the vertical growth [15].

The majority of patients, considered in this study, were hypoplastic AI type, which constituted 54%. A portion of 35.4% constituted hypomatured type and 10.6% the hypocalcified type. The above ratios were similar to ratios found in other studies [14, 16].

In this research, all types of dentition, primary, mixed, and permanent were encountered among AI patients.

In this literature review, it was not possible to evaluate AI prevalence with respect to gender as most patients' gender was not mentioned. However, Adorno-Farias et al. had confirmed that AI affects both genders equally [5].

Prevalence of dental caries among AI population was found to be low in the majority of the articles. This low rate of dental caries was linked to a lack of proximal contacts and elimination of fissures through enamel loss [17].

The low prevalence of dental caries for AI patients leads to wonder about dental caries specificity.

According to studies conducted on hypoplastic AI, the low rate of dental caries among AI patients were explained by the following phenomena.

- The salivary pH, which is significantly higher for hypoplastic AI patients compared to a control group, inhibits the carious process. In addition, streptococci strains are sensible to alkaline pH [18–20].
- The oral microbiome of the saliva showed a low rate of streptococci, which is a cariogenic bacteria [21]
- The weak cariogenic *Lactobacillus*, present in the microflora strains in AI hypoplastic patients, has an effect on the inhibition of *Streptococcus* [22–24]. Also, a significantly high level of *Bacillus* spp., *Enterococcus faecalis*, and *Enterococcus faecium* were found in AI hypoplastic patients, and these are proposed to serve as probiotics in oral health. [25–27].
- In vitro studies performed by Kammoun et al. [21] showed a significantly high adhesion of *Lactobacillus* and a weak adhesion of *Streptococcus mutans* on AI dental hard tissues

The study from Sundell et al. revealed that the susceptibility of caries is less prominent for the hypomineralized type of AI and severe cases of hypoplastic AI [10]. This could be explained by the fact that enamel quality differs among AI types. Further studies would allow a deeper understanding of the relationship between AI type and caries susceptibility.

In this literature review, it was noticed that few teeth of AI patients had to be extracted due to dental caries. These were mainly primary teeth which can be correlated with tooth exfoliation. This low rate of teeth extraction due to caries can be explained by the fact that dental caries in this type of population were not decaying. For this reason and according to Brosnan et al. guidelines, the need of extraction is rare as other treatment techniques are possible [28].

Dental filling due to caries was not highlighted in most examined articles. However, a big majority of these articles have focused on ways to improve esthetics and functional rehabilitation using different materials providing a deeper understanding of their behavior on the affected dental tissues.

Amalgam: all restorations with amalgam have been replaced as they were judged inadequate, as seen in the studies from Seow et al. and Markovic et al. [9, 17]. Therefore, considering its disadvantages, amalgam is far from being the material of choice for treating dental caries among AI patients [29].

Glass ionomer cement seems to be an acceptable choice of treatment. However, it remains a provisional solution [7, 9, 30].

Composite resin seems to be the material of choice. However, a problem of bond strength was encountered [31].

- Bond strength to abnormal enamel was noticed to be lower compared to normal enamel [32]. This was attributed by Pousette et al. to abnormal prism structure [7]. It was shown that direct composite restorations survival has been higher for hypoplastic form of AI than for hypomineralized AI. Faria-e-Silva et al. demonstrated a linear relationship between the hardness of enamel and bond strength [33]. To overcome this, some studies suggested the deproteinization procedure with 5% sodium hypochlorite prior to adhesive restoration as a solution to enhance bond strength. However, other studies found that such a procedure does not provide a significant improvement [34, 35].
- Bond strength to dentin is also weak for AI patients as dentin among AI patients gets affected. This is seen as thickening of the peritubular dentin, partial obliteration of the dentin tubules, and an increase of calcium levels particularly among teeth with hypocalcified AI. This morphological appearance reminds the sclerotic form of dentin, and it is thought to be highly acid resistant [33, 36, 37].

The weak bond strength, noted in dental tissue affected by AI, may be the reason for the occurrence of dental caries in 3 patients seen in this study who had performed previous bonding [6, 14, 37]. The low bond strength had probably provoked a leakage which promoted bacteria invasion.

### 4.1. DMF Index

Studies like those from Patel et al. and Pousette et al. hinted that AI patients are more susceptible for carious lesion than healthy patients [7, 38]. Other studies, including this one, demonstrated that this could not be valid in many situations. For example, it was reported in Adorno and colleague's study that the number of teeth with caries of Chilean patients with AI was similar to that found in healthy 12 years old Chilean children [5]. In fact, DMFT averages in some countries, such as India, Turkey, or Serbia, are higher compared to DMFT averages in Switzerland, New Zealand, and Sweden [39].

From the various data outlined in the results of this literature review, it can be noticed that DMFT averages of AI population depends on demographic parameters. For example, DMFT index of Turkish and Serbian AI patients, found in studies from Koruyucu et al. and Markovic et al. [8, 9], could be integrated in DMFT averages of healthy population of their respective countries [39]. In fact, the majority of the patients who had moderate or severe cariogenic process come from India and Turkey. Also, according to AlQobaly et al., smoking and oral hygiene appeared to be the variables that showed a consistent and significant association with coronal and root caries [40]. Patients from undeveloped countries generally have bad oral hygiene. Moreover, tooth sensitivity seen with AI patients in these countries tends to make things even worse [41].

Additionally, some studies such as the one from Bernabé et al. showed that caries prevalence could be linked to age. DMFT tends to increase with age and worsen in adulthood [42]. This study shows that this phenomenon also exists for AI population. Indeed, some patients in this study with severe or moderate caries fall in adulthood.

## 5. Conclusion

This study showed the following.

- There is a low incidence of dental caries and a low DMF index among AI population
- The combination of bacteria seen in the microflora of AI patients seems to give AI patients protection against dental caries
- The incidence of dental caries among AI population depends on sociodemographic factors. Prevalence of dental caries increases because of a lack of oral hygiene seen among patients in undeveloped countries. Also, DMFT index tends to increase with age.
- Eventhough dental filling due to dental caries was not highlighted in this study, composite resin seems to be the material of choice
- Few extractions due to dental caries were mentioned probably due to the low rate of caries in AI patients

## Figures and Tables

**Table 1:** Search strategy.

Search details	Number of articles
“Amelogenesis Imperfecta” [Mesh] and “Dental Caries” [Mesh]	25
“Amelogenesis Imperfecta” [Mesh] and “Tooth Loss” [Mesh]	1
“Amelogenesis Imperfecta” [Mesh] and “Dental Restoration, Permanent” [Mesh]	67
“Amelogenesis Imperfecta” [Mesh] and “DMF Index” [Mesh]	3
Total number of articles	96

## Data Availability

No data were used to support this study.
